# Optical properties and color stability of a translucent zirconia: effects of finishing after grinding

**DOI:** 10.1590/0103-6440202406094

**Published:** 2024-12-16

**Authors:** Pablo Machado Soares, Camila Pauleski Zucuni, Bruna Dias Ilha, Leticia Borges Jacques, Liliana Gressler May, Luiz Felipe Valandro

**Affiliations:** 1 Post-graduate Program in Oral Science, Prosthodontic Unit, Faculty of Odontology, Federal University of Santa Maria(UFSM), Santa Maria, Rio Grande do Sul, Brazil; 2 Prosthodontics Unit, Faculty of Odontology, Federal University of Santa Maria, Santa Maria, Rio Grande do Sul State, Brazil

**Keywords:** Color difference, roughness, translucency, zirconia

## Abstract

This study evaluated the effect of grinding and finishing treatments (polishing or glaze) on the color stability, translucency and opalescence of a translucent zirconia (3Y-TZP) after immersion in red wine. Discs (Ø= 12 mm; thickness 0.8 mm) of 3Y-TZP (Zenostar T, Ivoclar AG) were randomly allocated (n= 8) according to the surface treatment factor: Ctrl - as-sintered; Gr - grinding with diamond bur #4219; Gr + Pol - grinding followed by polishing; Gr + Gl - grinding followed by glaze. Surface roughness and scanning electron microscopy were carried out. All specimens were subjected to daily immersions in red wine for 30 minutes during 18 days. The color differences (ΔE_00_) were measured prior, after 9 and 18 days of immersion through the CIEDE2000 formula, as same as translucency (TP_00_) and opalescence (OP) parameters. Kruskal Wallis, repeated measures ANOVA/Tukey, and Spearman’s correlation tests were performed to evaluate the roughness, optical and correlation data, respectively. The Gr + Gl depicted the lowest ΔE_00_ value after 9 days. The Gr group showed the greatest ΔE_00_ values in all immersion times, whereas the other groups presented similar behavior after 18 days. The Gr and Gr + Pol groups had the highest Ra and Rz values. The correlation between roughness and color change was moderate and significant (p= 0.02, r= 0.42). No differences were observed for TP_00_ and OP. The surface treatments affected the roughness and color stability of zirconia, being glaze application more effective to reduce color alterations after the immersion protocol.

## Resumo

O objetivo foi avaliar o efeito de tratamentos de desgaste e acabamento (polimento ou glaze) sobre a estabilidade da cor, translucidez e opalescência de uma zircônia translúcida (3Y-TZP) após imersão em vinho tinto. Discos (Ø = 12 mm; espessura de 0,8 mm) de 3Y-TZP (Zenostar T, Ivoclar AG) foram aleatoriamente distribuídos (n=8) de acordo com o fator de tratamento de superfície: Ctrl - apenas sinterizado; Gr - desgaste com ponta diamantada #4219; Gr + Pol - desgaste seguido de polimento; Gr + Gl - desgaste seguido de glaze. Testes de rugosidade superficial e microscopia eletrônica de varredura foram realizados. Todos os espécimes foram submetidos a imersões diárias em vinho tinto por 30 minutos durante 18 dias. As diferenças de cor (ΔE_00_) foram medidas antes, após 9 e 18 dias de imersão através da fórmula CIEDE2000, assim como os parâmetros de translucidez (TP_00_) e opalescência (OP). Testes de Kruskal Wallis, ANOVA de medidas repetidas/Tukey e correlação de Spearman foram realizados para avaliar a rugosidade, dados ópticos e correlações, respectivamente. O grupo Gr + Gl apresentou o menor valor de ΔE_00_ após 9 dias (p˂0,05). O grupo Gr mostrou os maiores valores de ΔE_00_ em todos os tempos de imersão (p˂0,05), enquanto os outros grupos apresentaram comportamento semelhante após 18 dias. Os grupos Gr e Gr + Pol tiveram os maiores valores de Ra e Rz. A correlação entre rugosidade e mudança de cor foi moderada e significativa (p=0,02, r=0,42). Não foram observadas diferenças para TP_00_ e OP. Os tratamentos de superfície afetaram a rugosidade e a estabilidade de cor da zircônia, sendo a aplicação de glaze mais eficaz para reduzir as alterações de cor após o protocolo de imersão.

## Introduction

The use of all ceramic zirconia restorations has been increasing for oral-rehabilitations, considering the exponential demand for aesthetic and mimicking of natural teeth. In this context, second (3Y-TZP) and third-generation stabilized zirconia (4YSZ and 5YSZ), has been indicated to manufacture monolithic restorations, since the higher stabilizer and cubic crystals promote an improved translucency level for the polycrystalline ceramic ^1^. Even so, these materials may experience some phase transformation from tetragonal to monoclinic (t-m) when subjected to extrinsic stimulus, such as grinding or polishing, for instance ^2^. The (t-m) phase transformation generates a compressive tensile layer, which makes the crack propagation more difficult, thus increasing the mechanical properties and making zirconia an attractive restorative option from both the mechanical and aesthetical assumptions ^1^.

Regarding optical properties, even that 3Y-TZP exhibits high opacity when compared to other ceramics considering its small zirconium oxide crystals which produce a high light refraction index ^1^, monolithic restorations of this material may still be recommended in conservative thickness, mainly in posterior region. However, it is essential that the restoration keeps its color for a long time, since the success of treatment is associated not just to the mechanical longevity but also to the patient's satisfaction. A ceramic’s color stability can be affected by intrinsic factors such as composition and microstructure, as well as extrinsic factors such as dietary habits, food and oral hygienic agents ^3^ ^,^ ^4^. In addition, clinical discoloration can be caused by commonly consumed beverages such as coffee, tea, red wine and orange juice ^5^ ^,^ ^6^ ^,^ ^7^.

According to previous studies, color alterations can also be associated with texture and surface roughness generated by surface treatments ^3^ ^,^ ^8^. In this sense, clinical adjustments with discs or diamond burs are commonly performed to improve the marginal adaptation, the axial profile, and the proximal/occlusal contacts ^8^ ^,^ ^9^. However, this procedure can remove the surface glaze, leaving a rough surface capable of absorbing more pigments and accumulating biofilm, besides modifying light interaction and color aspects ^8^ ^,^ ^9^ ^,^ ^10^. Furthermore, a rough surface may increase the wear of antagonist restoration/tooth ^11^.

Finishing and polishing procedures are recommended to reduce surface roughness and scratches created by grinding, in order to produce a smooth surface. The literature has demonstrated that the surface generated by grinding may be harmful to the restoration performance ^11^ ^,^ ^12^, thus performing finishing/polishing or glaze application procedures after grinding a zirconia surface is indicated to reestablish the smoothness and the shine of the restoration ^7^ ^,^ ^13^. Besides, different polishing protocols applied to ceramic systems are commonly associated to changes in both color and translucency parameters ^13^, and a previous study reported that the polishing protocol is effective to revert the zirconia pigmentation after immersion in coffee and wine ^14^. On the other hand, glaze application may also be considered as an alternative to prevent discoloration overtime by applying a vitreous layer over the restoration ^7^ ^,^ ^12^, thus improving the surface smoothness. Even so, there is still no consensus regarding color stability of zirconia ceramics and different optical properties when comparing the effect of polishing protocols and glaze application to prevent pigmentation. Hence, to compare different methods in a standard and controlled condition through an in vitro study is essential to help researchers to define the best protocol to increase polishing and reduce pigment retention.

Thus, considering the aforementioned concepts, the aim of the present study was to evaluate the effect of two surface treatments (polishing and glaze) after grinding on the surface characteristics, color stability, translucency and opalescence of a translucent zirconia after an extrinsic stimulus (immersion in red wine). The null hypotheses were that the different surface treatments have no influence on the ^1^ surface and ^2^ optical properties’ stability of zirconia.

## Materials and Methods

The materials adopted in the present study, their compositions and manufacturers are described in Box 1.

**Box 1:**
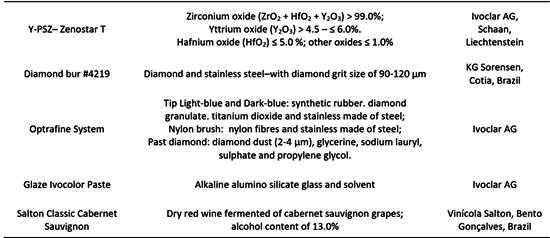
Description of materials, their composition and manufacturers.

Disc-shaped translucent yttrium-partially stabilized zirconia specimens (Y-PSZ - Zenostar T, Ivoclar AG) were obtained with final dimensions of Ø=12 mm in diameter and 0.8 mm in thickness. The pre-sintered zirconia blanks (Ø=98mm and 16mm in diameter) were sectioned in smaller blocks (14 mm × 14 mm) by the use of a diamond disc coupled to a hand-piece attached to an electric motor (Perfecta LA 623T, 1000-40,000 rpm - W&H, Bürmoos, Austria). Metal templates were attached to the blocks, which were rounded in a polishing machine (EcoMet/AutoMet 250, Buehler) to shape a 14 mm-diameter cylinder. Next, 1.2 mm-thickness slices were obtained using a cutting machine (Isomet 1000, Buehler) with a diamond disc under constant water cooling. After cutting, the discs were ground and polished on both sides with silicon carbide paper (SiC) with #1200-grit until reaching a thickness of 1 mm. The specimens were cleaned in an ultrasonic bath (1440 D, Odontobras) with 78% isopropyl alcohol for 5 minutes and then sintered according to the manufacturer’s instructions (1450°C for 2 h) in a specific furnace (Zyrcomat T 6000 MS, Vita Zahnfabrik). After that, the specimens were randomly assigned into 4 groups (n=8) according to the surface treatment to be performed (Control: just sintered; Grinding: just ground, with no following surface treatment; Polishing: grinding followed by polishing through a polishing kit; Glaze: grinding followed by glaze application), as described in Box 2.

**Box 2:**
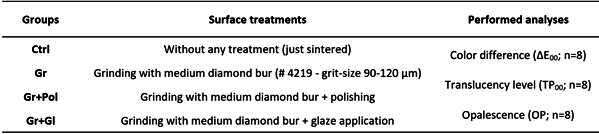
Experimental design.

### Surface treatments

### Grinding process

The specimens were cleaned in an ultrasonic bath (1440 D, Odontobras) with 78% isopropyl alcohol for 5 minutes and then a grinding protocol was performed with diamond bur (# 4219 - grit-size 90-120 μm, KG Sorensen) coupled with a force multiplier (T2 REVO R170 contra-angle handpiece up to 170,000 rpm, Sirona) in a low-speed motor (Kavo Dental) under constant water cooling (≈30 mL/min) and with oscillatory movements ^10^. The specimens were placed in a device so that the disc surface remained parallel to the diamond bur, and then a mark with pen was made on the entire surface of each specimen. The grinding process was performed until the mark was totally removed. One bur was used to grind every 2 specimens, and then was replaced by a new one.

### Polishing

For the polishing group, an OptraFine Polishing Kit (Ivoclar AG) consisting of 2 diamond rubbers (light blue and dark blue) and a nylon brush with a hand-piece coupled to a corresponding low speed motor (Kavo Dental, Biberach, Germany) a low-speed motor was used with a diamond paste and OptraFine HP Polishing Paste (Ivoclar AG), according to the experimental design.

### Glaze application

For the glaze group, a glaze material (Ivocolor glaze, Ivoclar AG) was applied over the ground surface according to the respective testing groups. The paste was mixed with the liquid until a homogeneous slurry was obtained in the consistency recommended by the manufacturer instructions. The mixture was applied with a brush on the surface of the specimen until a thin homogeneous layer was obtained. After drying, the specimens were fired in a specific furnace (Vacumat 6000 MP, Vita Zahnfabrik) according to the manufacturer’s recommendation (drying temperature 403°C, furnace closing time 6 min, heating rate 45°C/min, final temperature 710°C, maintenance of 1 min, with vacuum at 450°C and at 709°C).

### Surface roughness analysis

After the surface treatments, all specimens were analyzed on a specific roughness tester (Mitutoyo SJ-410, Mitutoyo Corporation, Kawasaki, Japan) according to the parameters of ISO 4287:1997 (Ra and Rz) to evaluate the obtained roughness pattern at each condition before the immersion protocol. The roughness evaluation method consisted of six measurements, being 3 on each axis (x and y) to obtain the Ra and Rz parameters. After that, mean values were obtained for each specimen.

### Immersion in red wine

After all surface treatments, the specimens were individually immersed in red wine (Salton Classic Cabernet Sauvignon, Vinícola Salton S.A.). The discs were immersed daily for 30 min in the wine at 37°C, and then brushed and washed in running water. After that, the specimens were dried and stored in distilled water for 24 hours until the next immersion protocol (Figure 2). This cycle was repeated for 18 days (total of 9 hours of immersion, corresponding to 6 months of consuming, according to Palla et al. (2018)) ^6^.

### Evaluation of optical properties stability

Optical properties were evaluated before immersion (baseline condition), then after 9 and 18 days of immersion. All specimens were washed in running water and dried prior to the color measurement to remove possible precipitates of the wine from the surface. The following parameters were analyzed: color difference (ΔE_00_), translucency (TP_00_) and opalescence (OP), comparing each condition of immersion with the baseline, which was considered as a reference parameter. Specimens were analyzed using an SP60 spectrophotometer (X-Rite, Grand Rapids) in analysis mode to obtain the CIEL*a*b* coordinates (Commission Internationale de l'Éclairage), with L* from 0 (black) to 100 (white), a* from green (−a*) to red (+a*), and b* from blue (−b*) to yellow (+b*). Each specimen was measured over a white (L*= 93.07, a*= -1.28, b*= 5.25), black (L*= 27.94, a*= - 0,01, b * = 0.03) (LENETA Card - model 12H - Color and Appearance) and gray backgrounds (L*= 50.30, a*=-1.41, b*= -2.37) (Mennon gray cards, Mennon photographic and technical Co.) ^15^. The L*, a*, and b* coordinate values were collected three times for all backgrounds in all the evaluation periods for each specimen, and the mean of those readings was used for statistical purposes. A drop of a coupling substance (glycerol C_3_H_8_O_3_) (Vetec Química Fina Ltda.) with a refractive index of 1.47 was used under the specimen to avoid dispersing light between the specimen and the spectrophotometer ^16^. The clinical thresholds described by Paravina et al. (2015) were considered in this study, where ΔE_00_ > 0.8 corresponds to the perceptibility limit and ΔE_00_ > 1.8 to the clinical unacceptability limit ^17^. Values obtained in the measurements on the gray background were used to calculate color difference (ΔE_00_) between the immersed and baseline specimens by the use of the CIEDE2000 formula (Equation 1):

$${\Delta E}_{00}={\left[{\left(\frac{∆L'}{K_{L}S_{L}}\right)}^{2}+{\left(\frac{∆C'}{K_{C}S_{C}}\right)}^{2}+{\left(\frac{∆H'}{K_{H}S_{H}}\right)}^{2}+R_{T}\left(\frac{∆C'}{K_{C}S_{C}}\right)\left(\frac{∆H'}{K_{H}S_{H}}\right)\right]}^{\frac{1}{2}}$$( eq. 1 )

In which: ΔL’, ΔC’, and ΔH’ are the differences in lightness, chroma, and hue, respectively, for a pair of measurements and RT is a function (the so-called rotation function) which accounts for the interaction between chroma and hue differences in the blue region. Weighting functions S_L_, S_C_, and S_H_ adjust the total color difference for variation in the location of the color difference pair in L’, a’, b’ coordinates, and the parametric factors k_L_, k_C_, and k_H_ are correction terms for deviation from reference experimental conditions. In the present study, the parametric factors of the CIEDE2000 color difference formula were set as 1.

The same formula was adopted to evaluate the values obtained in the measurement on the black and white backgrounds were used to determine the translucency of the specimens through the translucency parameter (TP_00_), where a higher number indicates higher translucency.

Perceptibility and acceptability thresholds described by Salas et al. (2018) were considered to evaluate the translucency difference, where ΔTP_00_ > 0.62 corresponds to the perceptibility limit and ΔTP_00_ > 2.62 to the clinical unacceptability limit ^18^.

Opalescence (OP) is described as the ability to reflect blue when white light strokes the material, and was calculated through the values of the coordinates a’ and b’ which were used on the white (W) and black (B) backgrounds ^19^.

$$OP=\sqrt{\left(a_{B}^{*}-a_{W}^{*})+(b_{B}^{*}-b_{W}^{*}\right)}$$ ( eq. 2 )

In which: ΔL’, ΔC’, and ΔH’ are the differences in lightness, chroma, and hue, respectively, for a pair of measurements and RT is a function (the so-called rotation function) which accounts for the interaction between chroma and hue differences in the blue region. Weighting functions S_L_, S_C_, and S_H_ adjust the total color difference for variation in the location of the color difference pair in L’, a’, b’ coordinates, and the parametric factors k_L_, k_C_, and k_H_ are correction terms for deviation from reference experimental conditions. In the present study, the parametric factors of the CIEDE2000 color difference formula were set as 1.

The same formula was adopted to evaluate the values obtained in the measurement on the black and white backgrounds were used to determine the translucency of the specimens through the translucency parameter (TP_00_), where a higher number indicates higher translucency.

Perceptibility and acceptability thresholds described by Salas et al. (2018) were considered to evaluate the translucency difference, where ΔTP_00_ > 0.62 corresponds to the perceptibility limit and ΔTP_00_ > 2.62 to the clinical unacceptability limit ^18^.

Opalescence (OP) is described as the ability to reflect blue when white light strokes the material, and was calculated through the values of the coordinates a’ and b’ which were used on the white (W) and black (B) backgrounds ^19^.

### SEM evaluation

The surface topography of all groups after the surface treatments was performed by Scanning Electron Microscopy (SEM - Vega3, Tescan, Czech Republic) under 5000 × magnification. To do so, two specimens per group were cleaned in an ultrasonic bath (1440 D - Odontobras) with 78% isopropyl alcohol for 5 min, and then gold sputtered before the topographical analysis.

### Statistical analysis

The data normality and homoscedasticity were verified using the Shapiro-Wilk and Levene tests, respectively. Repeated measures two-way analysis of variance was used to analyze ΔE_00_, TP_00_ and OP after the immersion protocols. All pairwise multiple comparisons were conducted using Tukey’s test for optical properties. The Kruskal-Wallis test was used to analyze the Ra and Rz parameters since the roughness data followed a non-normal distribution. The correlation between ΔE_00_ and Ra was performed using Spearman’s Correlation (α = 0.05). All data were analyzed by the Statistica 7.0 statistical software program (Stat Soft. Inc.).

## Results

The roughness and color difference results are depicted in Table 1. The surface treatment was determinant for the roughness values (p ˂ 0.001), since the Gr and Gr + Pol groups presented the highest Ra and Rz values, which were statistically similar between them. On the other hand, the Ctrl presented the lowest values of Ra and Rz, followed by the Gr + Gl.

**Table 1:** Color difference (ΔE_00_) means (Standard Deviation) and surface roughness (Ra and Rz) medians (Quartiles 25% and 75%) of each group.

Groups	Color differences from baseline condition		Roughness analysis	
	ΔE_00_ 9 Days	ΔE_00_ 18 Days	Ra (Q1 - Q3)	Rz (Q1 - Q3)
Ctrl	1.19 (0.30)^Bb^	1.34 (0.40)^Bb^	0.17 (0.16 - 0.20)^c^	1.30 (1.23 - 1.76)^c^
Gr	2.65 (0.36)^Ba^	2.94 (0.34)^Ba^	2.36 (1.78 - 2.47)^a^	13.20 (11.26 - 14.10)^a^
Gr+Pol	1.29 (0.35)^Bb^	1.65 (0.65)^ABb^	1.34 (0.99 - 1.77)^ab^	8.33 (5.54 - 9.72)^ab^
Gr+Gl	0.65 (0.11)^Cc^	1.66 (0.46)^Bb^	0.48 (0.44 - 0.71)^b^	2.54 (2.31 - 3.19)^bc^

Within rows, different capital letters denote statistically significant differences, while lowercase letters within columns reveal statistically significant differences (p<0.05)

Regarding to the color stability after immersion, the repeated measures two-way analysis of variance and multiple comparisons showed that both surface treatment and immersion protocol factors affected the color difference values (p ˂ 0.001). Considering the adopted thresholds, the Ctrl and Gr + Pol groups showed detectable but also acceptable ΔE_00_ values after 9 and 18 days when compared to the baseline condition (1.8 > ΔE_00_ > 0.8). Gr group showed the greatest ΔE_00_ values in all immersion times, which were considered clinically unacceptable after the immersion for both 9 and 18 days (ΔE_00_ > 1.8). The Gr + Gl group depicted the most stable color pattern when compared to the baseline condition after 9 days of immersion (ΔE_00_ ˂ 0.8), which was increased after 18 days to a detectable but also acceptable value of ΔE_00_. Correlation between roughness and color difference was moderate and significant (p = 0.02, r = 0.42).

The results of translucency and opalescence are depicted in Tables 4 and 5, respectively. Both optical properties were not statistically affected by the factors under study (translucency - p = 0.180; opalescence - p = 0.259). However, considering the thresholds for translucency difference (ΔTP_00_) it can be noticed that the Gr and Gr + Gl were the most stable groups after immersion, followed by the Ctrl and Gr + Pol groups. For opalescence, similar values were observed after all periods of immersion of all specimens, regardless of the performed surface treatment.

**Table 2:** Translucency parameter (TP_00_) means (Standard Deviation) of different groups on different evaluation times.

Groups	Translucency Parameter				
	Baseline	9 Days	18 Days	ΔTP_00_ B-9 days	ΔTP_00_ B-18 days
Ctrl	17.64 (4.04)^Aa^	16.05 (2.80)^Aa^	17.92 (1.70)^Aa^	1.59	0.28
Gr	17.06 (3.54)^Aa^	17.60 (0.75)^Aa^	16.92 (0.80)^Aa^	0.54	0.14
Gr+Pol	19.31 (1.43) ^Aa^	16.71 (2.32)^Aa^	17.43 (1.25)^Aa^	2.60	1.88
Gr+Gl	18.13 (3.00) ^Aa^	18.00 (2.15)^Aa^	18.31 (1.34)^Aa^	0.13	0.18

Within rows, different capital letters denote statistically significant differences, while lowercase letters within columns reveal statistically significant differences (p<0.05)

**Table 3:** Opalescence parameter (OP) means (SD) of different groups on different evaluation times.

Groups	Opalescence Parameter		
	Baseline	9 Days	18 Days
Ctrl	10.93 (2.12)^Aa^	10.03 (1.59)^Aa^	10.75 (0.98)^Aa^
Gr	10.92 (2.09)^Aa^	10.90 (0.42)^Aa^	10.63 (0.33)^Aa^
Gr+Pol	12.17 (0.84)^Aa^	10.81 (1.49)^Aa^	10.90 (0.64)^Aa^
Gr+Gl	11.13 (1.70) ^Aa^	11.04 (1.17)^Aa^	11.19 (0.82)^Aa^

Within rows, different capital letters denote statistically significant differences, while lowercase letters within columns reveal statistically significant differences (p<0.05)

**Figure 1:**
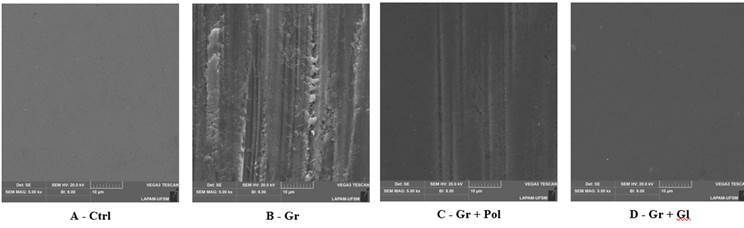
Representative SEM images (1000× magnification) of Y-TZP surface. It noticed that grinding (Gr) produces a rough surface with scratches and flaws; the polishing and glaze depicted a potential to reduce such alterations.

SEM images (Figure 1) showed an irregular topography for the Gr group, which was rougher and presented more scratches and grooves. Such defects were reduced after polishing (Gr + Pol group), even so some residual grooves were still noticed. The images demonstrate smoother and regular surfaces for the Control and Gr + Gl groups.

## Discussion

As mentioned, intrinsic and extrinsic factors can affect the light interaction and color of dental ceramics ^3^ ^,^ ^4^ ^,^ ^8^. Defects as grain boundaries and pores are intrinsic factors which can interfere in translucency and opacity ^20^. The cement layer, material thickness and LTD (low-temperature degradation) can be cited with respect to extrinsic factors ^4^, as same as the exposition to liquids ^21^, which are present in acidic beverages such as coffee, tea, red wine, cola and fruit juices ^5^ ^,^ ^6^ ^,^ ^21^. The drinks mentioned above present low pH, which is responsible for the color change ^5^ ^,^ ^6^. In fact, these sentences are in accordance with the findings of the present study, since it was noticed that both the roughness and topographical aspect after grinding was modified, as same as the glaze application and the immersion protocol in red wine affected the color differences for zirconia. Thus, the null hypotheses were rejected.

The grinding group showed higher surface roughness (Ra) and relevant color difference values (ΔE_00_) rather than the other groups, being the only condition with unacceptable color changes after 9 days of immersion in red wine. This finding can be explained by an increase in surface roughness and defects introduced by grinding with a diamond bur (Figure. 1, Table 1). It was previously stated that the surface defects generate higher discoloration than a smooth surface ^3^. In addition, an irregular textured surface reflects less amount of light than a smooth surface, as well as generating an irregular and diffuse light reflection pattern ^8^ ^,^ ^13^. In this sense, the literature corroborates that treatments such as grinding, polishing and glazing can affect the color stability of ceramics in time, since these procedures alter the texture, surface roughness and retention of pigment ^3^ ^,^ ^8^. From that viewpoint, the findings corroborate that an additional clinical procedure is essential after the use of diamond burs on the zirconia surface.

According to a previous study, glaze application seems to be the most suitable approach to produce higher color stability ^7^, which was corroborated by our findings, since the found color difference was considered irrelevant when comparing 9 days of immersion and the baseline condition (Table 1). After 18 days, the color difference was detectable, however it was considered acceptable according to the adopted threshold ^17^. On the other hand, the grinding process followed by a polishing protocol was not as effective as glaze application to maintain the color stability after either 9 and 18 of immersion in red wine. Zucuni et al. (2017) showed that glaze layer generates smoother surfaces when compared with finishing/polishing ^12^, which results in less pigment retention from foods and drinks ^6^ ^,^ ^7^, thus explaining our findings. The obtained SEM images also corroborate the results, showing that the polishing protocol was less effective than glaze to revert the grooves and defects generated by grinding (Figure 1). Thus, it seems clear that the glaze application over a ground surface may be the best choice to promote a regular surface and reduce pigment incorporation over time.

Regarding the translucency, it can be characterized as the property which allows light passage and dispersion, thereby making the material more opaque or transparent ^22^. In the present study, no statistical differences were observed between all conditions, and all translucency differences were considered at least clinically acceptable when considering the thresholds cited by Salas et al. (2018) (0.62 for perceptibility and 2.62 for acceptability) ^18^. However, some conditions showed perceptible differences after the immersion in red wine. It was previously reported that the surface roughness may affect the translucency of dental materials due to the scattering effect ^23^. Indeed, the translucency stability was higher for the grinding and grinding + glaze groups, while the higher translucency difference was observed in the grinding + polishing group, followed by the control group. A previous study demonstrated that polishing generated a higher decrease in translucency in zirconia than glaze application ^24^, which is in accordance with our findings. Thus, the smooth surface and the presence of a vitreous layer after the glaze application may explain such maintenance of the optical behavior. Even so, such mentioned differences were considered small, and all translucency differences were satisfactory.

Another important optical property is opalescence, which is represented by differences between the transmitted and reflected light ^25^. In this study, the opalescence was considered statistically similar for all evaluated conditions, indicating that such property was kept stable after the immersion in red wine (Table 3). These findings are in accordance with the quantitative analysis performed for the translucency parameter, which showed a stable behavior for this property. However, it must be considered that no threshold was adopted for the opalescence, since to the authors knowledge there is no indicator for such optical property. Even so, it may be considered that the light interaction was not affected in a critical level after the immersion protocol for both translucency and opalescence.

Despite the relevant findings of the present study, their limitations must also be considered in order to give directions for future studies on this thematic. Only one staining solution was considered for this study, and consequently the exposure was to only one pH value during the immersion protocol, which is different from an individual’s diet, which consists in the sum of several solutions. However, the adopted methodology was important to evaluate the optical properties in a standard and controlled scenario, thus increasing the validity of the obtained findings. Finally, it must be considered that the absence of resin cement in the present evaluation is a limitation, since the cement may also be affected by pigments. Thus, future studies should consider different scenarios as other solutions such as coffee and juice, as same as the use of cemented ceramics and glaze materials usually applied in clinical practice.

## Conclusion

Within the limitations of this current study, it was concluded that:

- Grinding with a diamond bur induces a rougher surface and increases the color difference caused by wine.
- The glaze application promoted a smooth surface and more color stability for the zirconia ceramic.
- The translucency and opalescence properties of the zirconia ceramic were stable after the immersion protocol, regardless of the surface treatment.
